# 

**Published:** 1880-01

**Authors:** 


**TERMS.—50 Cents a Year. Address The Bistoury, Elmira, N. Y.**

### SUBSCRIPTION RECEIVED.

TO JANUARY, 1880.

In this column we will credit every subscription receive during the last quarter. Subscribers will please notify us immediately if their payments are not duly credited. The State only is given—this, with the full name of the party subscribing, will be sufficient to indicate who is meant.

NEW YORK—Dr Wm H Green, R Morris, D W Andrews, Henry Roberts, Col J H Horton, E W Finch, Dr Jno Armstrong, Dr H B Smith, Dr Seymour Davis, Dr L B Harrington, Dr J H Robinson, Dr Abel Mineere, Dr O B Kingsington, Dr Jas Harris, Dr D Fordham, Dr N D Leets, Dr Amos Snyder, Mrs B W Hines, Mrs Anna Lathrop, Dr N Faulkner, J M Eustice, Dr Ellwanger, Dr H R Harrison, Mrs D M Green, Oscar Hines, L V Mercereau, Dr J Kingsley, Dr Sara Boynton, Dr Forest Willard, Dr A A Root, Dr Lesley Combs, Dr P J Lawhead, Jas D Stiles, S M Sayles, Mrs F A Seymour, Dr H Epps, Dr Z Offenheimer, Dr H M Paine.

PENNSYLVANIA—Dr Wm Cable, W G Shoemaker, C S Knight, Dr Wm C Coleman, Dr Sam Hollenstien, Dr O B Arol, Dr John Snyder, Dr R B Backus, Dr Geo Gumberling, Dr F D Wood, Dr S M Ames, Samuel Loring, A A Schoch, Mrs D Wentz, Dr H Harper, Dr N Jessup, Dr Dan Waggenseller, Dr Harvey Smith, Dr Geo Gunterman, Mrs J D Shindel, Mrs Lucy James, Dr. O. Leisenring, Chas. Peters, Samuel L Northrup, Dr Jno Snyder, D D Loring, Mrs D F Bishop, Dr C H Norwood.

ALABAMA—Dr J Erickson, Mrs O Combs, Dr D Butts.

FLORIDA—Dr H J Hammond, Dr N D Sweiss.

GEORGIA—Dr A Forseyth, Dr Jno Kirkpatrick, Mrs D Flavius.

ILLINOIS—Mrs D Chambers, Dr D D Jessup, Dr A B Howe, Dr Sam Ketchum, Dr L D Clark, Dr M L Christainy, Mrs S B Thomas, Dr J Hansom, F D Hisom.

IDAHO—Mrs W D Bigtow.

INDIANA—Dr H D Welles, Dr Jos Dodd, Dr A Samuels, Mrs J M Harrison, F F Fields, Dr Snow.

KENTUCKY—John Banks, Hiram Smith, Dr L M Snook.

MINNISOTA—Dr C Wiensma.

MASSACHUSETTS—Dr T H Manley, Dr J G Harrio, Dr A M DeWitt, Dr S D Martain, Mrs Jno Eberly, H M Ransom.

OHIO—Dr Samuel Ralston, Dr Geo Cook, Dr D D Weise, Mrs E Savage, Mrs N A Haverly, Mrs D Coons, W Virg, Dr A Roberts.

WISCONSIN—Mrs Jane McComber, Dr Chas Freeman, Mrs Dr Rice.

NO NAME—One Dollar from Quincey Ill.

PRINTED FOR THE PROPRIETOR, AT THE DAILY ADVERTISER JOB PRINTING HOUSE.
ELMIRA, N. Y.

